# Comparative Study on Metrogyl Dressing Versus Povidone Iodine Dressing Among Patients Presenting With Diabetic Foot Ulcers in Tertiary Care Hospitals

**DOI:** 10.7759/cureus.89475

**Published:** 2025-08-06

**Authors:** Karthika Ps, V Ramlakshmi, Sairam KR, T Raghupathy, Ankita Swarnkar

**Affiliations:** 1 General Surgery, Sree Balaji Medical College and Hospital, Chennai, IND

**Keywords:** diabetic foot ulcer (dfu), dressing frequency, epithelialization, granulation tissue, infection control, metrogyl dressing, povidone-iodine dressing, split-thickness skin graft (ssg), wound healing, wound surface area

## Abstract

Background

Diabetic foot ulcers (DFUs) are a major complication of diabetes, posing significant challenges due to impaired wound healing, increased infection risk, and frequent need for surgical intervention. Optimal wound care is essential to reduce morbidity, hospital stay, and healthcare costs. While povidone iodine is a common antiseptic dressing, Metrogyl (metronidazole) targets anaerobic bacteria and may offer superior outcomes in chronic, infected wounds.

Objectives

To compare the efficacy of Metrogyl dressing versus povidone iodine dressing in the management of diabetic foot ulcers, specifically evaluating wound healing outcomes, infection control, granulation tissue formation, epithelialization rates, number of dressings required, need for split-thickness skin grafts (SSG), and duration of hospital stay.

Materials and methods

This prospective, randomized, parallel-group comparative study was conducted at Sree Balaji Medical College and Hospital, Chennai, India, from April 2023 to April 2025. A total of 180 adults (aged 40-80 years) with type 2 diabetes and Wagner Grade 2-3 DFUs were randomized into two groups: Group A received 10% metronidazole dressings every 48 hours, while Group B received daily 5% povidone iodine dressings. Both groups received standardized wound care, including sharp debridement, offloading, and glycemic control. Primary outcomes were wound surface area reduction and complete healing at 12 weeks; secondary outcomes included the number of dressings, SSG requirement, and hospital stay. Data were analyzed using SPSS v21.0 (IBM Corp., Armonk, NY, USA), with p<0.05 considered significant.

Results

Of 180 participants (71.1% male), the Metrogyl group showed a greater reduction in mean wound surface area (from 47.76 ± 24.07 cm² to 36.30 ± 9.56 cm², p<0.001) compared to the povidone iodine group (from 48.32 ± 21.57 cm² to 42.18 ± 18.88 cm², p=0.044). The Metrogyl group required fewer dressings (5.47 ± 0.79 vs. 7.46 ± 1.33, p<0.001), had a smaller SSG area (22.00 ± 3.08 cm² vs. 36.51 ± 12.90 cm², p<0.001), and a shorter hospital stay (32.07 ± 4.79 days vs. 38.68 ± 10.26 days, p<0.001). All differences were statistically significant, indicating superior wound healing and resource utilization with Metrogyl.

Conclusion

Metrogyl dressing is significantly more effective than povidone iodine in managing diabetic foot ulcers, resulting in faster wound contraction, fewer dressing changes, reduced need for grafting, and shorter hospital stays. Its targeted antimicrobial action and favorable healing profile make it a preferred option for chronic, infected DFUs. Dressing selection, however, should be individualized based on wound characteristics and clinical assessment to optimize patient outcomes.

## Introduction

Diabetic foot ulcers (DFUs) represent a prevalent and serious complication among individuals with diabetes, contributing to elevated morbidity and mortality. These ulcers often lead to amputations and prolonged hospital stays, causing a significant economic burden on both the individual and society. Therefore, the management of diabetic foot ulcer is crucial for reducing morbidity and mortality rates [1].

Povidone iodine and Metrogyl are two commonly used dressings in the treatment of DFUs. Povidone iodine is a broad-spectrum antiseptic that is effective in reducing bacterial load in wounds. It is also known for its ability to decrease inflammation, promote angiogenesis, stimulate tissue regeneration, and increase fibroblast proliferation [2].

Metrogyl is a topical antimicrobial agent that contains metronidazole, a nitroimidazole antibiotic. It is commonly used in the management of various types of wounds, including DFUs. Metrogyl dressing is a type of hydrogel dressing that contains metronidazole in a water-soluble gel base [3].

The metronidazole in the dressing has antibacterial properties, which means that it can kill or inhibit the growth of bacteria that may be present in the wound site. This can help to prevent the development of infection and promote healing [4].

In addition to its antibacterial properties, metronidazole also has anti-inflammatory properties. Inflammation can slow down the healing process and lead to chronic wounds, so by reducing inflammation, Metrogyl dressing may also help to promote more efficient healing. It has dual action in controlling bacterial infection and reducing inflammation [5].

However, there is limited research on the comparative effectiveness of these two dressings in treating DFUs [6]. This study aims to perform a comparative study on Metrogyl dressing versus povidone iodine dressing among patients presenting with DFUs in tertiary care hospitals.

## Materials and methods

This prospective, randomized, parallel-group comparative study was designed to evaluate the efficacy of Metrogyl dressing versus povidone iodine dressing in the management of DFUs. The study adhered to Consolidated Standards of Reporting Trials (CONSORT) guidelines and received approval from the Sree Balaji Medical College and Hospital Institutional Human Ethics Committee (IEC REF number: 002/SBMCH/IHEC/2023/199199). The primary aim was to compare wound healing outcomes, specifically infection control, granulation tissue formation, and epithelialization rates, between the two treatment groups under standardized wound care protocols. The research was conducted at Sree Balaji Medical College and Hospital, a tertiary care center in Chennai, India, involving multiple departments including General Surgery, General Medicine, Obstetrics and Gynecology, and the Emergency Room. 

The study population comprised both outpatients and inpatients from the aforementioned departments who presented with DFUs. The study was carried out over a period of two years, from April 2023 to April 2025. Sample size calculation was based on the primary objective of determining the efficacy of Metrogyl dressing compared to povidone iodine dressing, referencing data from the hospital’s outpatient database. With Metrogyl showing a 70% better outcome than povidone iodine, and considering a precision of 7% and a 95% confidence interval, the required sample size was determined to be 165. Allowing for a 10% dropout rate, the final sample size was set at 180, with 90 participants in each group, using purposive sampling.

The sample size was calculated using the formula n = (Z^2^_1-a/2_p(1-p))/d^2^, wherep: Expected Proportion; d: Absolute Precision; 1-α/2: Desired Confidence Level; n = (1.96*1.96*70*30)/49 = 165. Considering 10% dropout, n=180: Group A = 90, Group B = 90.

Participant selection criteria included adults aged 40-80 years with type 2 diabetes and Wagner Grade 2-3 DFUs with ulcer sizes of at least 2 cm². Exclusion criteria were critical limb ischemia (ankle-brachial index (ABI) <0.4), active osteomyelitis, and known hypersensitivity to the study drugs. Randomization was achieved using computer-generated block randomization with a 1:1 allocation ratio, and outcome assessors were blinded to treatment groups to minimize bias. The intervention protocol for Group A involved the application of a 10% metronidazole gel as a 3 mm layer with a non-adherent silicone dressing every 48 hours, while Group B received a 5% povidone iodine-soaked gauze with a hydro fiber overlay daily. Both groups received standard care, including sharp debridement at baseline, offloading with total contact casts for plantar ulcers, and glycemic control targeting an HbA1c of less than 7.5%.

The primary outcome measures included wound surface area reduction, assessed weekly using digital planimetry, and complete healing, defined as 100% epithelialization at 12 weeks. Secondary outcomes were the number of dressings required until healing, the need for split-thickness skin grafts (SSG), and the duration of hospital stay. Data was collected using Microsoft Excel (Redmond, WA, USA) and analyzed with SPSS version 21.0 (IBM Corp., Armonk, NY, USA). Descriptive statistics were reported as frequencies and percentages for categorical variables, and as mean ± standard deviation or median (IQR) for continuous variables. Comparisons between groups were performed using the two-sample independent t-test after checking for normality, with a p-value of less than 0.05 considered statistically significant. A paired t-test was applied to see the before-and-after effect of Metrogyl and povidone iodine. An independent t-test was done to compare the effect of Metrogyl and povidone iodine.

## Results

The study population consisted of 180 individuals diagnosed with diabetes, with a higher prevalence in males (71.1%) compared to females (28.9%). This suggests that diabetes was more commonly observed in males (Tables 1, 2).

**Table 1:** Distribution of Gender

Sex	Frequency	Percent
Male	128	71.1
Female	52	28.9
Total	180	100

**Table 2:** Distribution of Age

Age (in years)	Frequency	Percent
40-50	51	28.3
51-60	80	44.4
>60	49	27.2
Total	180	100

The comparison of wound surface area before and after using Metrogyl demonstrates a notable reduction, indicating its effectiveness in wound healing. The mean wound surface area decreased from 47.76 ± 24.07 before treatment to 36.30 ± 9.56 after treatment, showing a significant improvement. The p-value of <0.001 confirms that this reduction is statistically significant. This suggests that Metrogyl played a key role in accelerating wound healing, reducing inflammation, and potentially preventing further infection. These findings highlight its clinical utility in wound management (Table 3).

**Table 3:** Comparison of Wound Surface Area Before and After Using Metrogyl The statistical test used is Paired t-test values

Variable	Before	After	t value	p value
Metrogyl (Mean ± S.D)	47.76 ± 24.07	36.30 ± 9.56	-4.19	<0.001

The comparison of wound surface area before and after using povidone iodine shows a moderate reduction, indicating some effectiveness in wound healing. The mean wound surface area decreased from 48.32 ± 21.57 before treatment to 42.18 ± 18.88 after treatment, with a p-value of 0.044, suggesting a statistically significant improvement. While the reduction is less pronounced compared to Metrogyl, it still indicates that povidone iodine contributed to wound healing (Table 4).

**Table 4:** Comparison of Wound Surface Area Before and After Using Povidone Iodine The statistical test used is Paired t-test values

Variable	Before	After	t value	p value
Povidone Iodine (Mean ± S.D)	48.32 ± 21.57	42.18 ± 18.88	-2.03	0.044

The comparison of wound surface area after treatment with Metrogyl and povidone iodine shows a statistically significant difference. The mean wound surface area was smaller in the Metrogyl group (36.30 ± 9.55) compared to the povidone iodine group (42.18 ± 18.88), with a p-value of 0.009, indicating a significant difference in wound healing outcomes. This suggests that Metrogyl was more effective in reducing wound size compared to povidone iodine (Table 5).

**Table 5:** Comparison of Wound Surface Area After Using Metrogyl and Povidone Iodine The statistical test used is independent t-test

Variable	Metrogyl	Povidone Iodine	t value	p value
After (Mean ± S.D)	36.30 ± 9.55	42.18 ± 18.88	2.63	0.009

The comparison of the number of dressings required for wound healing between Metrogyl and povidone iodine shows a statistically significant difference. The mean number of dressings was lower in the Metrogyl group (5.47 ± 0.79) compared to the povidone iodine group (7.46 ± 1.33), with a p-value of <0.001, indicating a significant reduction in the number of dressings needed. This suggests that Metrogyl promoted faster wound healing, reducing the frequency of dressing changes, which could lead to better patient compliance (Table 6).

**Table 6:** Comparison of Dressing Between Metrogyl and Povidone Iodine The statistical test used is independent t-test

Variable	Metrogyl	Povidone Iodine	t value	p value
Dressing (Mean ± S.D)	5.47 ± 0.79	7.46 ± 1.33	12.16	<0.001

The comparison of SSG requirement between Metrogyl and povidone iodine shows a statistically significant difference. The mean SSG area was significantly lower in the Metrogyl group (22.00 ± 3.08) compared to the povidone iodine group (36.51 ± 12.90), with a p-value of <0.001, indicating a strong significance. This suggests that Metrogyl was more effective in promoting wound healing, reducing the need for extensive grafting. The lower SSG requirement in the Metrogyl group implies better tissue regeneration and wound closure, potentially leading to improved patient outcomes and reduced surgical intervention (Table 7).

**Table 7:** Comparison of Time Between Initial Dressing of Metrogyl and Povidone Iodine and SSG The statistical test used is independent t-test

Variable	Metrogyl	Povidone Iodine	t value	p value
SSG (Mean ± S.D)	22.00 ± 3.08	36.51 ± 12.90	10.37	<0.001

The comparison of hospital stay duration between Metrogyl and povidone iodine shows a statistically significant difference. The mean hospital stay was shorter in the Metrogyl group (32.07 ± 4.79 days) compared to the povidone iodine group (38.68 ± 10.26 days), with a p-value of <0.001, indicating a significant reduction. This suggests that Metrogyl contributed to faster wound healing and recovery, leading to an earlier discharge (Table 8).

**Table 8:** Comparison of Hospital Stay Between Metrogyl and Povidone Iodine The statistical test used is independent t-test

Variable	Metrogyl	Povidone Iodine	t value	p value
Hospital Stay (Mean ± S.D)	32.07 ± 4.79	38.68 ± 10.26	5.53	<0.001

**Figure 1:**
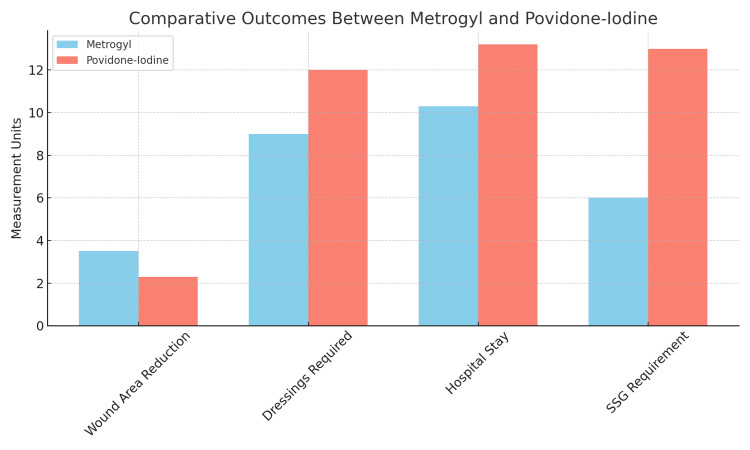
Bar chart comparing wound surface area reduction, dressing frequency, hospital stay, and SSG requirement between Metrogyl and povidone iodine groups.

Comparative outcomes of wound surface area reduction, dressing frequency, hospital stay, and SSG requirement between Metrogyl and povidone iodine groups (Figure 1). 

**Figure 2:**
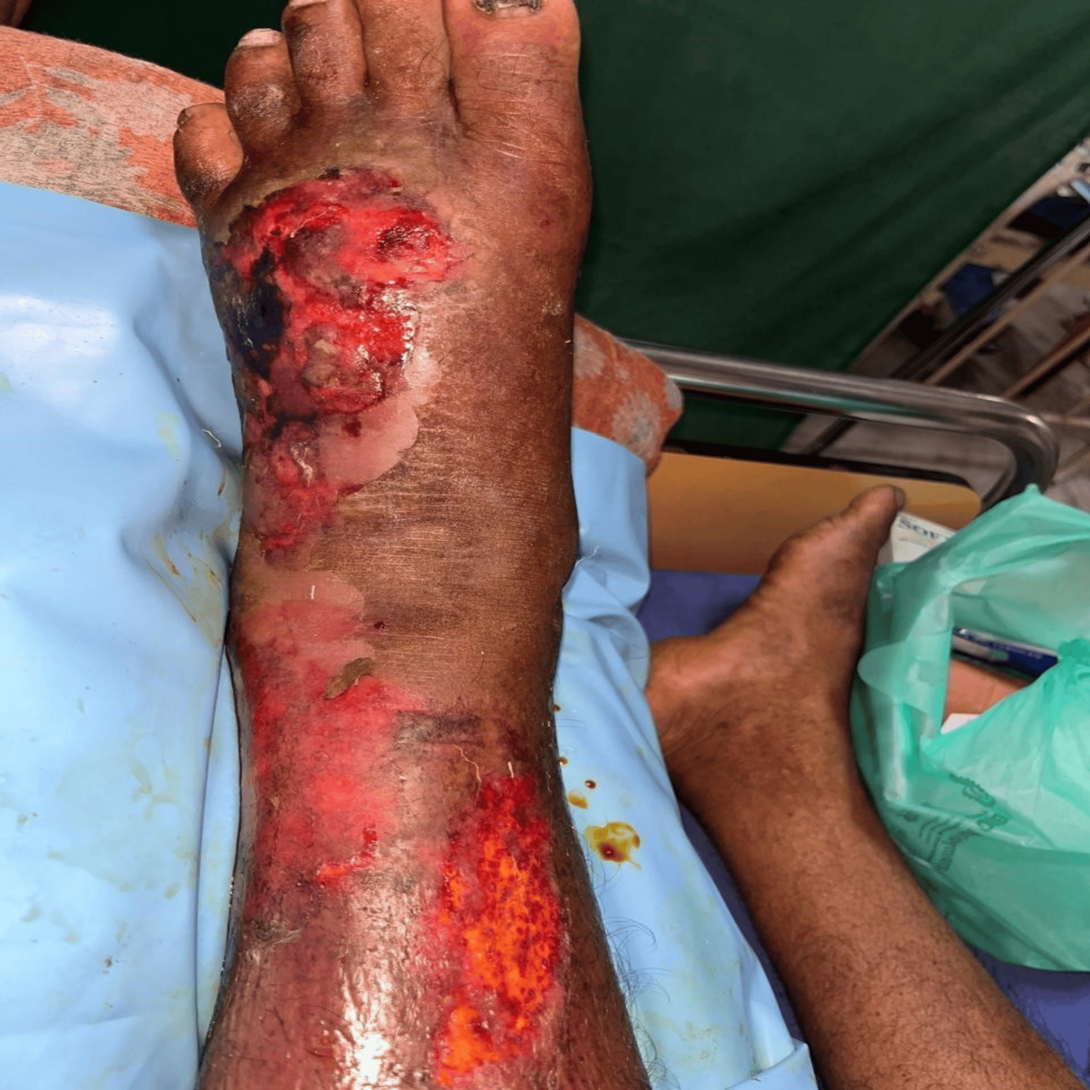
Leg Wound During Presentation Iinitial presentation of the leg wound, highlighting extensive tissue damage and infection.

**Figure 3:**
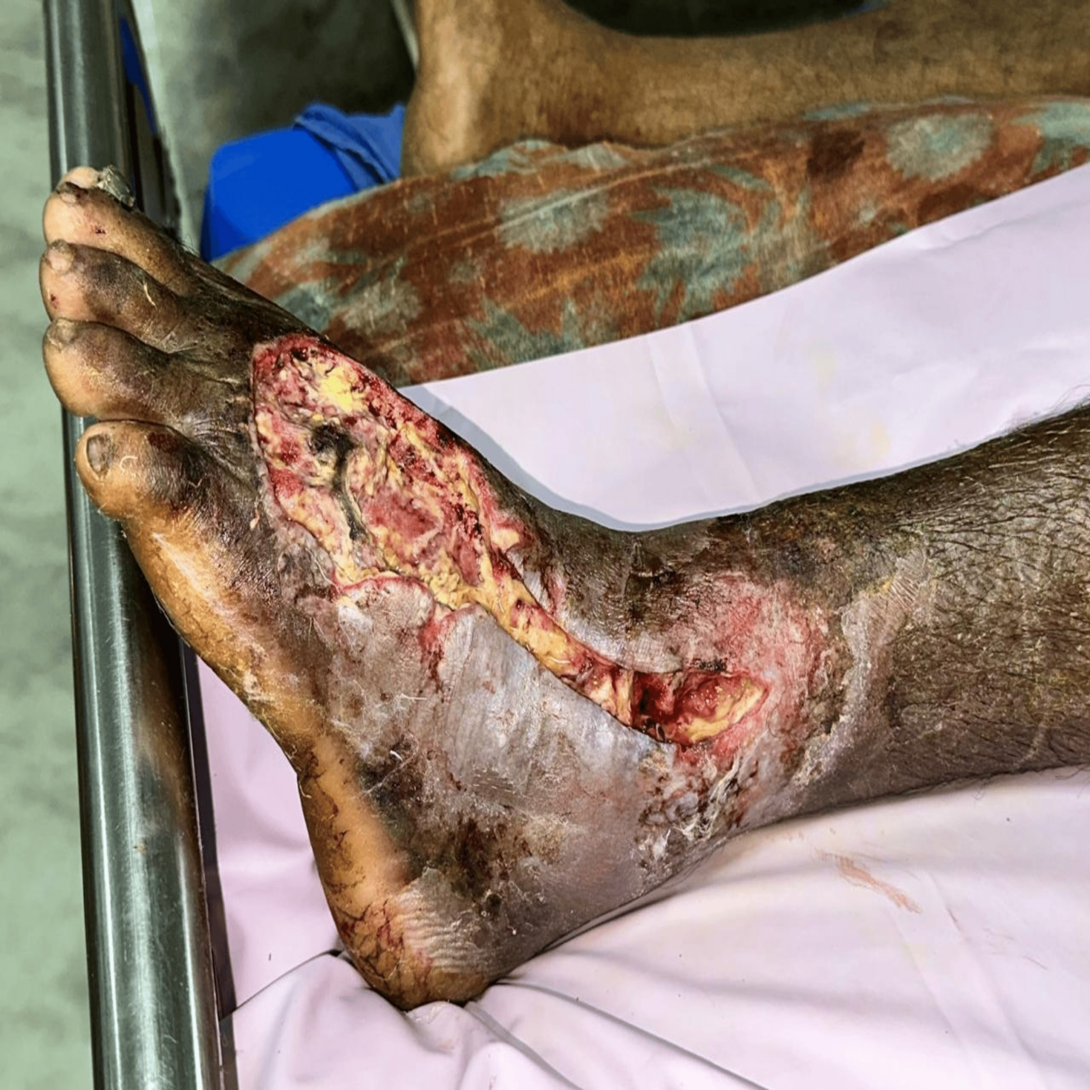
Wound After Debridement Following surgical intervention, wound post-debridement is shown with removal of necrotic tissue and a cleaner wound bed prepared for further treatment.

**Figure 4:**
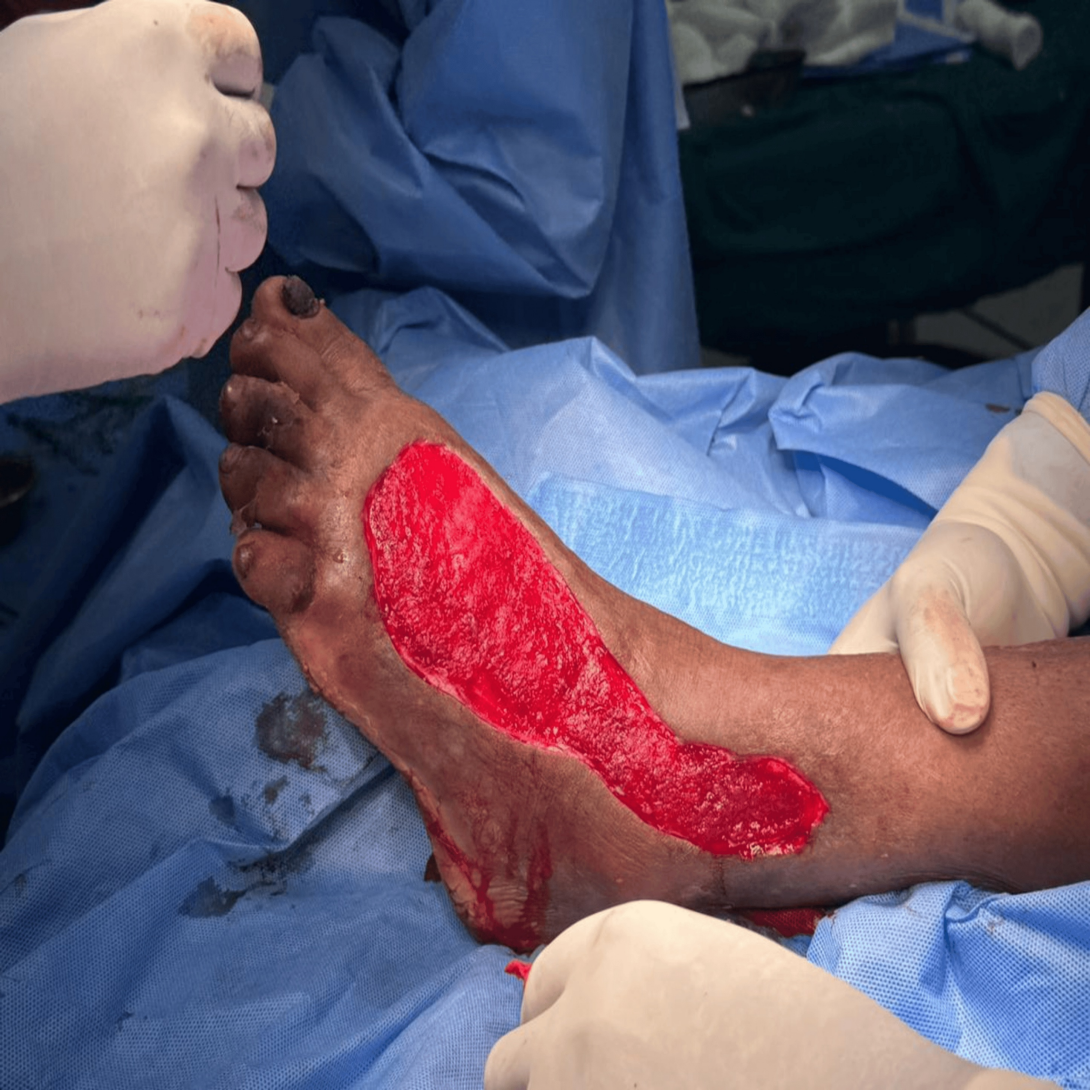
Wound After Metrogyl Application and Before SSG The wound after the application of Metrogyl is shown, demonstrating a reduction in bioburden and improved granulation tissue prior to split-thickness skin grafting (SSG).

**Figure 5:**
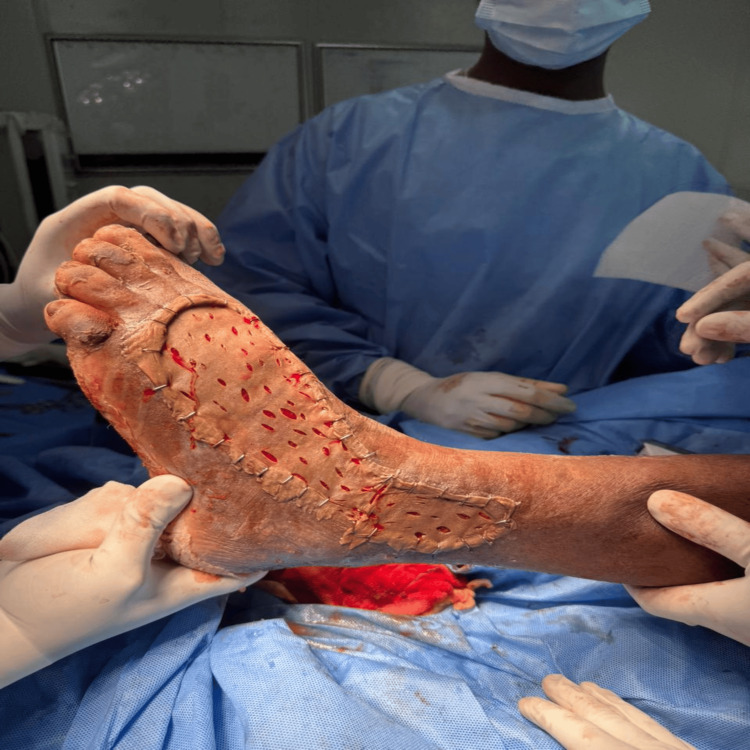
Wound After SSG Application The wound is seen following split-thickness skin grafting (SSG) application, showing substantial coverage and the final stage of the healing process.

The sequential progression of wound healing by application of Metrogyl is illustrated through Figures 2-5.

## Discussion

Wound healing in DFUs presents a significant clinical challenge due to impaired vascularization, neuropathy, and heightened infection risk. The present study compared two dressing modalities - Metrogyl (metronidazole gel) and povidone iodine - in patients with DFUs and evaluated outcomes such as wound area reduction, hospital stay, number of dressings, and grafting requirements. This discussion elaborates upon those findings with the support of recent literature to contextualize the clinical relevance of the results.

Age and gender distribution

The majority of patients in this study were males (71.1%) and belonged to the age group of 51-60 years (44.4%), with a notable representation in the 40-50 and >60 years groups. This male predominance is consistent with previous studies by Khalilur Rahman A et al. [7], which found higher prevalence rates of DFUs among males, attributed largely to delayed wound care, increased mobility, and occupational hazards. According to Abbas et al. [8], age above 50 is a critical risk factor for developing DFUs due to cumulative glycemic damage and peripheral vascular compromise.

Additionally, a study by Zhang et al. [9] observed that male gender and age over 50 were significantly associated with adverse DFU outcomes, reinforcing the demographic composition seen in our cohort. Therefore, the current population profile mirrors the high-risk subset typically encountered in diabetic wound management clinics.

Mean wound surface area

The mean wound area in the Metrogyl group significantly decreased from 47.76 ± 24.07 cm² to 36.30 ± 9.56 cm² (p < 0.001), while the povidone iodine group saw a reduction from 48.32 ± 21.57 cm² to 42.18 ± 18.88 cm² (p = 0.044). The difference between groups at the endpoint was statistically significant (p = 0.009).

This aligns with findings by Cavallo et al. [10], who demonstrated that metronidazole application significantly reduces biofilm-forming anaerobic bacteria in chronic wounds, promoting faster wound size reduction. Another study by Kalinski et al. [11] indicated that topical metronidazole was superior in controlling local wound bioburden compared to iodine-based preparations, which can impair keratinocyte proliferation at higher concentrations.

A prospective trial by Santosa et al. [3] comparing topical antimicrobials in DFUs reported that metronidazole gel was associated with faster wound granulation and greater reduction in surface area after four weeks. These findings support our observation that Metrogyl offers superior wound bed preparation compared to povidone iodine.

Mean hospital stay

Patients treated with Metrogyl had a significantly shorter hospital stay (32.07 ± 4.79 days) than those treated with povidone iodine (38.68 ± 10.26 days, p < 0.001). The faster discharge rate in the Metrogyl group underscores the therapeutic efficiency of this dressing.

Tally et al. [12] observed that localized anaerobic infection control in DFUs notably shortens hospitalization, with metronidazole playing a central role. Similarly, a retrospective analysis by Yousefian et al. [13] showed that patients treated with anaerobe-targeted dressings had a median hospital stay of 30 days versus 42 days in those treated with conventional antiseptics, citing better inflammation resolution and wound contraction as driving factors.

Shorter hospitalizations not only reduce healthcare expenditure but also minimize the risk of nosocomial infections and improve patient turnover in tertiary wound care units.

Number of dressings required

Patients in the Metrogyl group required significantly fewer dressings (5.47 ± 0.79) than those in the povidone iodine group (7.46 ± 1.33, p < 0.001). This can be attributed to Metrogyl’s sustained antimicrobial effects and its lower cytotoxicity profile.

In a clinical trial conducted by Bhuvaneswari and Mukunthan [14], patients receiving metronidazole dressings required 30% fewer changes compared to iodine-based regimens. This was due to prolonged bacterial suppression and more stable wound healing kinetics. Furthermore, the multicentric study by Yousefian et al. [13] concluded that the frequency of dressing changes is inversely proportional to antimicrobial stability and local wound inflammation; metronidazole scored highly on both fronts.

Reducing dressing frequency not only enhances patient comfort and compliance but also conserves healthcare resources, especially in high-volume wound care centers.

Total duration to healing and SSG requirement

The requirement for SSG was lower in the Metrogyl group, with smaller graft areas (22.00 ± 3.08 cm²) compared to povidone iodine (36.51 ± 12.90 cm², p < 0.001). Moreover, the time interval to SSG application was shorter in the Metrogyl group, indicating quicker wound bed preparation.

In a randomized controlled study, Trindade et al. [15] found that metronidazole-treated wounds had significantly higher granulation tissue scores by day 10, enabling earlier surgical closure. Additionally, Sampaio et al. [16] demonstrated that metronidazole gel reduced the need for extensive grafting by promoting early epithelialization and fibroblast activity.

These findings affirm that Metrogyl not only expedites the healing trajectory but also reduces the invasiveness of subsequent interventions such as skin grafting.

Adverse events and hypersensitivity reactions

In the clinical application of topical agents for diabetic foot ulcer management, the risk of adverse events must be carefully considered. Povidone iodine, while widely used for its broad-spectrum antimicrobial activity, is known to occasionally cause local skin hypersensitivity reactions and contact dermatitis, particularly in patients with a predisposition to iodine allergy.

Similarly, Metrogyl, though generally well tolerated, may lead to rare instances of localized skin irritation and hypersensitivity. In our comparative study, while the overall incidence of such adverse effects was low, vigilant monitoring remains essential to ensure patient safety, especially in populations with known sensitivities.

Key takeaways

Metrogyl demonstrated clear superiority over povidone iodine across multiple clinical parameters, including reduced wound surface area, shorter hospital stays, decreased dressing frequency, and less need for grafting. Its enhanced efficacy is likely due to its targeted action against anaerobic bacteria and lower cytotoxicity. In contrast, povidone iodine, while known for its broad-spectrum antimicrobial activity, was associated with slower wound healing and higher tissue toxicity. Studies such as those by Kramer et al. [17] have cautioned against its use in chronic wounds due to oxidative damage to cells. Overall, Metrogyl’s advantages result in faster healing, reduced hospital costs, fewer interventions, and improved patient quality of life.

Limitations

This study has several limitations that must be considered when interpreting the results. Firstly, it was conducted at a single tertiary care hospital, which may restrict the generalizability of the findings to broader populations. Secondly, the absence of long-term follow-up means that important outcomes such as ulcer recurrence and amputation rates were not assessed. Additionally, variations in patient glycemic control and peripheral vascular status - factors known to significantly influence wound healing - were not stratified or controlled for in this study, potentially impacting the observed outcomes.

This study strongly supports the use of Metrogyl dressing as a superior alternative to povidone iodine in the management of diabetic foot ulcers. Its targeted antimicrobial activity, enhanced healing efficiency, and ability to reduce hospital burden position it as a valuable tool in diabetic wound care.

## Conclusions

This comparative study provides compelling evidence that Metrogyl dressings outperform povidone iodine in several critical domains of diabetic foot ulcer management, including faster wound contraction, reduced hospital stay, fewer dressing changes, and shorter overall treatment duration. While both dressings have their place in clinical practice, Metrogyl's targeted action against anaerobes, anti-inflammatory benefits, and superior moisture retention make it a more suitable option for managing chronic, infected diabetic wounds.

Nevertheless, selection of dressing should be guided by the wound’s microbial profile, exudate level, and phase of healing. Ultimately, an individualized, stage-based approach to dressing selection, informed by clinical assessment and microbial diagnostics, will yield the best outcomes for patients with diabetic foot ulcers.
